# Comment on “A jumbo cyanophage encodes the most comprehensive ribosomal protein set in the known virosphere” by Isaac Meza-Padilla *et al*.

**DOI:** 10.1093/ismejo/wrag241

**Published:** 2026-09-30

**Authors:** LinXing Chen

**Affiliations:** State Key Laboratory of Advanced Environmental Technology, School of Environment, University of Science and Technology of China, Hefei, Anhui, 230026, China

**Keywords:** jumbo phage, cyanophage, ribosomal protein, prophage remnant, mobile genetic element

Meza-Padilla *et al*. recently reported that the jumbo phage genome PhiMa05 encodes six ribosomal proteins (RPs), a ribosome biogenesis protein, and an RP S18-alanine *N*-acetyltransferase, and interpreted this genome as the most comprehensive set of RPs identified in viruses to date [1]. This is an intriguing claim with potentially broad implications for how microbial ecologists interpret the boundary between viral and cellular genetic repertoires. However, the evolutionary interpretation of this study is based on a prerequisite assumption that PhiMa05 is indeed an intact jumbo cyanophage genome. Our independent analyses suggest that this assumption requires substantial re-evaluation.

PhiMa05 was originally reported as a lytic jumbo phage infecting *Microcystis*, with a linear double-stranded (ds) DNA genome of ~274 kb (GenBank accession number: MW495066.1) [2]. Meza-Padilla *et al*. reanalyzed this genome and focused on its RP complement [1]. We agree that several predicted proteins in this genome are confidently annotated as RPs or RP-associated proteins. The presence of RPs is therefore not, by itself, evidence against a viral origin, given that virus-encoded RPs have been reported previously [3]. However, the issue we raise here is whether the larger genome context of PhiMa05 supports its interpretation as an intact virion-forming jumbo phage.

To address this question, we analyzed the PhiMa05 genome using independent virus-identification and structure-based annotation approaches. Both viral prediction tools, geNomad [4] and VirSorter2 [5], classified the PhiMa05 genome as non-virus in our analyses. Because any single viral prediction tool may miss unusual or highly divergent viruses, this result alone should not be considered definitive. However, it becomes important when combined with structural and gene-content evidence. We predicted the protein structures for all PhiMa05-encoded genes using ColabFold [6] and compared the resulting models against several reference structure databases. This analysis recovered only a limited phage-like structural signal. In particular, we also detected a putative major capsid protein and a phage portal protein (Fig. 1). These proteins are most similar to structures of proteins from relatively small phages, consistent with the structural comparisons noted in the original article (Supplementary Fig. S1 in Meza-Padilla *et al*. [1]). However, we did not recover a coherent virion morphogenesis and DNA-packaging module expected for an intact tailed jumbo phage genome. This distinction is critical. A complete dsDNA tailed phage genome is expected to encode a set of coordinated hallmark functions, typically including a terminase large subunit, phage portal protein, major capsid protein, head maturation or head-completion proteins, tail or tail-associated proteins, and often lysis-related genes. In the PhiMa05 genome, the evidence appears very limited. We did not identify a recognizable large terminase or DNA-packaging system or a complete head–tail morphogenesis module.

**Figure 1:**
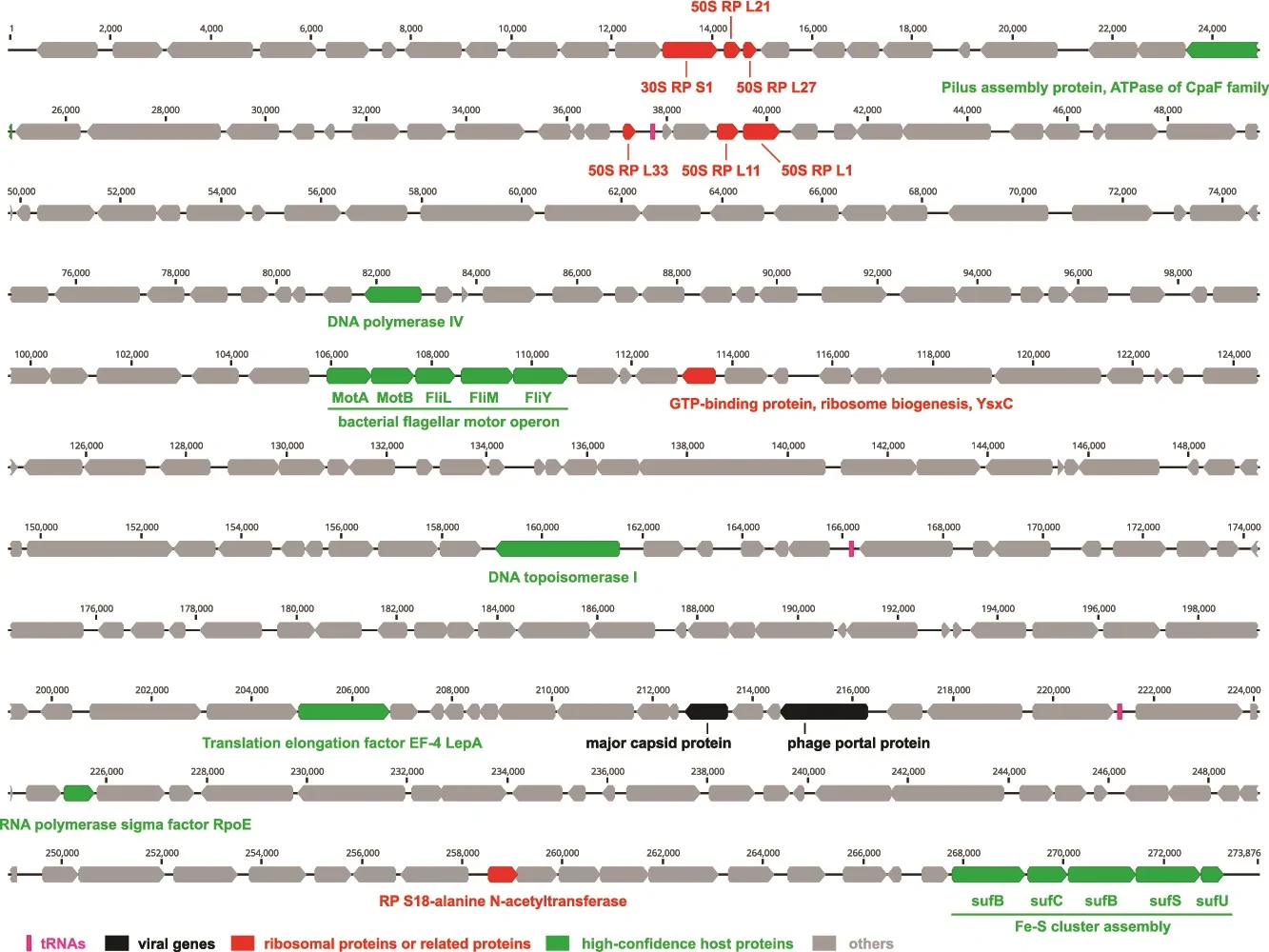
Functional annotation of the protein-coding genes encoded by the PhiMa05 genome; the annotations are based on protein structures (predicted by Colabfold) and confirmed by an online Basic Local Alignment Search Tool for proteins (BLASTp) search for associated functional domains (see details in Supplementary Information S1); all confidently identified viral genes are shown; a subset of metabolism-related genes that could help to determine the genetic origin is shown here; via the online BLASTp, we confirmed that the DNA polymerase IV, DNA topoisomerase I, RNA polymerase sigma factor RpoE, and translation elongation factor EF-4 LepA are more closely related to those from bacteria instead of viruses.

Furthermore, the broader gene-content context reinforces this concern. The genome encodes many proteins more typical of cellular life. For example, a bacterial flagellar motor-associated gene cluster (*MotA, MotB, FliL, FliM*, and *FliY*), a CpaF-family pilus assembly ATPase, as well as an Fe-S cluster assembly system. Such features are not impossible in mobile genetic elements (MGEs) such as phages that have mixed ancestry, but their abundance contrasts with the absence of a coherent phage structural and packaging module. Moreover, the DNA polymerase IV, DNA topoisomerase I, RNA polymerase sigma factor RpoE, and translation elongation factor EF-4 LepA encoded by PhiMa05 are most closely related to those from Bacteria. Thus, the detected major capsid and phage portal proteins are more parsimoniously interpreted as a localized prophage-remnant-like signal or a phage-derived island within a larger non-viral genetic element, rather than evidence that the entire 274-kb PhiMa05 genome represents an intact jumbo cyanophage.

It is notable that prophage remnants containing phage portal and major capsid protein genes are not rare in nature. For example, in a *Candidatus Melainabacteria* bacterium genome reconstructed from a landfill sample, we identified a scaffold (GenBank accession number: JBFXCH010000002.1; 358 677 bp) with a phage portal protein gene and a major capsid protein gene that shared 60% and 81% amino-acid identity with the corresponding PhiMa05 proteins, respectively (Fig. 2). This homologous region likewise lacks other recognizable genes required for complete phage morphogenesis and a DNA-packaging module.

**Figure 2:**
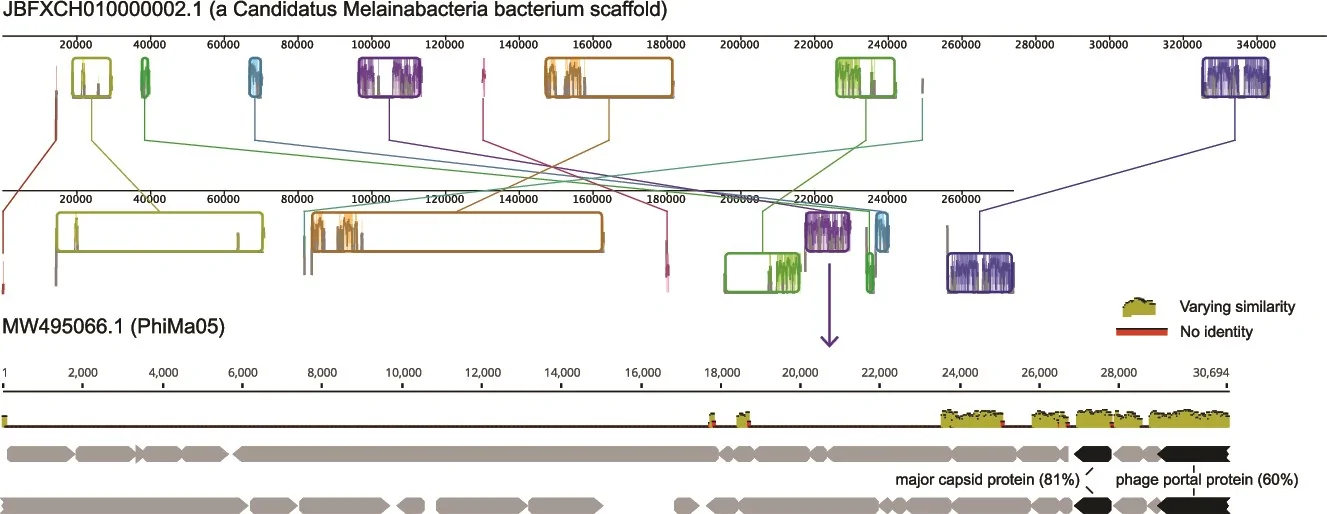
Similar major capsid protein and phage portal protein in a *Candidatus Melainabacteria* bacterium genome; the whole genome alignment of the *Candidatus Melainabacteria* bacterium genome and PhiMa05 is shown at the top; the vertical placement of the two tracks is arbitrary and is used only to separate the two genomes; it does not represent a quantitative variable; colored blocks indicate homologous genomic regions identified by pairwise sequence alignment; the purple block, including the major capsid protein and the phage portal protein, is zoomed in to show the details; the protein sequence identities are shown in brackets.

Together, our analyses provide a simpler explanation for the presence of bacterial-like ribosomal and ribosome-biogenesis genes, as well as the reported similarity between PhiMa05 regions and *Vampirovibrio* genomic regions. In the original article [1], these similarities were interpreted through a complex evolutionary scenario involving acquisition of RP genes by a PhiMa05 ancestor, integration into *Vampirovibrio*-like hosts, possible replacement of host copies, and subsequent host-range shift or expansion. If PhiMa05 is not an intact viral genome, however, much of this scenario becomes unnecessary or at least unsupported. We emphasize that our concern is not that viruses cannot encode RPs. They can, and documented cases have reshaped discussions of viral auxiliary functions and virus–host evolutionary exchange [3, 7, 8]. The concern is that a genome must first be confidently established as viral before its gene content is used to make claims about the limits of the virosphere. In the case of PhiMa05, the current combination of negative viral prediction results, absence of a complete structural and DNA-packaging module, and extensive cellular gene content does not support its classification as an intact jumbo cyanophage.

Importantly, our interpretation does not deny the original report of the phage particles or lytic activity associated with *Microcystis* [2]. Morphological observation can support the presence of a virus in the preparation, but it does not, by itself, establish that a particular assembled contig corresponds to the complete genome of that virion. The critical question is therefore not whether a phage was present, but whether the reported 274-kb PhiMa05 genome is directly linked to the observed particles. In cultures or lysates that may contain host-associated microorganisms, cellular DNA, or prophage-containing bacterial fragments can potentially be co-extracted and assembled together with, or instead of, the true virion genome. Viromics workflows typically reduce contamination from cellular DNA through filtration, nuclease treatment, and density-gradient purification prior to sequencing. Thus, one plausible explanation is that genuine phage particles were present in the original preparation, whereas MW495066.1 represents a co-purified bacterial fragment carrying a limited prophage-remnant region. Direct evidence connecting the 274-kb sequence to the observed particles would be needed to exclude this possibility. In principle, reanalysis and *de novo* assembly of the original raw sequencing reads would provide the most direct way to evaluate whether MW495066.1 reflects the true virion genome, host-derived contamination, or an assembly artifact. However, the raw sequencing reads do not appear to have been publicly deposited [2].

The issue we present here has broader relevance for microbial ecology. As metagenomic approaches increasingly reveal unexpected genes in viral datasets, the risk of overinterpreting cellular or composite MGEs as viruses becomes substantial [9]. RPs, metabolic, and other host-derived genes can be genuinely encoded by viruses, but they can also indicate host contaminations, misassembled regions, plasmids, integrative and conjugative elements, or prophage remnants. For unusual genomes that challenge conceptual boundaries between viruses and cells, viral identity should be established using multiple orthogonal lines of evidence, including but not limited to gene-content-based viral prediction, hallmark structural and DNA-packaging genes, genome architecture, assembly and coverage consistency, and, when possible, experimental validation from purified virions. This caution is also consistent with recent large-scale comparative analyses of cyanophage protein repertoires, which show that such unusual gene-content signals are rarely supported by conserved genomic context across distantly related viruses [10]. While our analyses question the current classification of PhiMa05 as an intact jumbo cyanophage, we acknowledge that commonly used viral identification and structural annotation tools may have limited sensitivity toward highly divergent or atypical phage genomes. Therefore, we cannot exclude the possibility that PhiMa05 represents an unusual divergent jumbo phage, and its evolutionary origin remains uncertain pending additional evidence. Resolving this point is essential not only for interpreting PhiMa05 but also for setting appropriate standards for future claims of ribosomal-protein-rich viruses.

## Supplementary Material

Supplmentary_information_wrag241

## Data Availability

The protein structure files and the structure comparison results against structure references are deposited at Figshare via https://doi.org/10.6084/m9.figshare.32600163.
