# Supplementary material for: Comment on “A jumbo cyanophage encodes the most comprehensive ribosomal protein set in the known virosphere” by Isaac Meza-Padilla et al
Source: ISME J. 2026 Sep 30;20(1):wrag241. doi: 10.1093/ismejo/wrag241 (PMC13623518; doi:10.1093/ismejo/wrag241)
Supplement: Supplementary file 1 [file supplmentary_information_wrag241.pdf]

Supplementary information

for

Comment on "A jumbo cyanophage encodes the most comprehensive ribosomal protein set in the known virosphere" by Isaac Meza-Padilla et al.

LinXing Chen [1,*]

[1] State Key Laboratory of Advanced Environmental Technology, School of Environment, University of Science and Technology of China, Hefei, China

[*] Corresponding author: LinXing Chen, linxingchen@ustc.edu.cn

Keywords: jumbo phage; cyanophage; ribosomal protein; prophage remnant; mobile genetic element

Supplementary methods

Protein structure prediction and annotation. The protein-coding genes of the jumbo phage genome were predicted for protein structures using ColabFold (downloaded on 22 November 2024) [1], with the following parameters: "--num-recycle 3, --use-gpu-relax, -- amber, --stop-at-score 70." For structure annotation, the predicted structures with a pLDDT score of ⩾70 were searched against the predicted structures of BFVD [2] and the NCBI protein families (NCBIfam) [3], using FoldSeek 9.427df8a [4] with the "easy-search" command (-c = 0.5, --cov-mode = 0). Only those hits with a TM score ⩾0.5 ("alntmscore") were retained for further analyses. The proteins annotated as viral or bacterial marker genes were manually confirmed individually using the online BLASTp search (https://blast.ncbi.nlm.nih.gov/Blast.cgi?PROGRAM=blastp).

Supplementary references

1. Kim, G. *et al.* Easy and accurate protein structure prediction using ColabFold. *Nat. Protoc.* **20**, 620–642 (2025).
2. Kim, R. S., Levy Karin, E., Mirdita, M., Chikhi, R. & Steinegger, M. BFVD-a large repository of predicted viral protein structures. *Nucleic Acids Res.* (2024) doi:10.1093/nar/gkae1119.
3. Camargo, A. P. *et al.* A genomic atlas of the human gut virome elucidates genetic factors shaping host interactions. *bioRxiv* (2025) doi:10.1101/2025.11.01.686033.
4. van Kempen, M. *et al.* Fast and accurate protein structure search with Foldseek. *Nat. Biotechnol.* **42**, 243–246 (2024).
